# Phospholipase D2 in prostate cancer: protein expression changes with Gleason score

**DOI:** 10.1038/s41416-019-0610-7

**Published:** 2019-11-01

**Authors:** Amanda R. Noble, Karen Hogg, Rakesh Suman, Daniel M. Berney, Sylvain Bourgoin, Norman J. Maitland, Martin G. Rumsby

**Affiliations:** 1 Cancer Research Unit, Department of Biology, University of York, York, YO10 5DD UK; 2 Technology Facility, Department of Biology, University of York, York, YO10 5DD UK; 3 Department of Molecular Oncology, Barts Cancer Institute, Queen Mary University of London, London, EC1M 6BQ UK; 4 Centre de Recherche du CHU de Québec, Axe des Maladies Infectieuses et Immunitaires, local T1-58, 2705 boulevard Laurier, Québec, G1V 4G2 QC Canada

**Keywords:** Prostate cancer, Cell growth, Time-lapse imaging

## Abstract

**Background:**

Phospholipases D1 and D2 (PLD1/2) are implicated in tumorigenesis through their generation of the signalling lipid phosphatidic acid and its downstream effects. Inhibition of PLD1 blocks prostate cell growth and colony formation. Here a role for PLD2 in prostate cancer (PCa), the major cancer of men in the western world, is examined.

**Methods:**

PLD2 expression was analysed by immunohistochemistry and western blotting. The effects of PLD2 inhibition on PCa cell viability and cell motility were measured using MTS, colony forming and wound-healing assays.

**Results:**

PLD2 protein is expressed about equally in luminal and basal prostate epithelial cells. In cells from different Gleason-scored PCa tissue PLD2 protein expression is generally higher than in non-tumorigenic cells and increases in PCa tissue scored Gleason 6–8. PLD2 protein is detected in the cytosol and nucleus and had a punctate appearance. In BPH tissue stromal cells as well as basal and luminal cells express PLD2. PLD2 protein co-expresses with chromogranin A in castrate-resistant PCa tissue. PLD2 inhibition reduces PCa cell viability, colony forming ability and directional cell movement.

**Conclusions:**

PLD2 expression correlates with increasing Gleason score to GS8. PLD2 inhibition has the potential to reduce PCa progression.

## Background

Many studies now implicate phospholipase D (PLD) in tumorigenesis since total PLD activity and the expression of its two major isoforms PLD1 and PLD2 are elevated in many cancers where increases can correlate with prognosis.^1–8^ Higher PLD activity is also linked to survival and migration signals in human breast cancer cells and in androgen-insensitive prostate cancer cell lines.^9,10^ Selective inhibition of PLD1 or PLD2 also makes breast cancer cells more sensitive to radiation.^11^ Investigations into the role of PLD in cancer have been aided by the development of new isoform-specific PLD1 and PLD2 inhibitors,^12–14^ which reduce the proliferation of breast cancer cells in mice.^7,12,14^

The link between PLD and tumorigenesis is through phosphatidic acid (PtdOH), a product of PLD1 and PLD2 activity.^15,16^ PtdOH is an intermediate in complex lipid synthesis^17^ but it is also a signalling lipid which, on formation, binds proteins at membrane surfaces leading to their activation.^13,18–20^ The involvement of PtdOH in the recruitment and activation of mTOR (mammalian target of rapamycin), Raf and Akt/PKB kinase has defined a role for PLD in regulating cell survival, proliferation and tumorigenesis.^19–21^ PtdOH formation also inhibits protein phosphatase 1 and upregulates the NFκB and Wnt signalling pathways, further promoting both cancer cell survival and metastasis.^21–23^

PLD2 is reportedly located at the plasma membrane under basal conditions,^24,25^ complexed with receptors in lipid rafts.^26,27^ This is in contrast to PLD1, which is localised to perinuclear membranes in cells^25,28,29^ but translocates to the plasma membrane on cell stimulation.^25,28^ PLD2 is also detected in the nucleus in a few reports.^4,28,30–32^ PLD2 is activated by protein kinase C (PKC)^33,34^ and by receptor (e.g. EGFR, PDGFR) and non-receptor (eg. Src, JAK3) tyrosine kinases^35,36^ while PLD1 is activated by PKC, casein kinase-II and small GTPases ARF and RHO.^21,37–39^ PLD2 has a higher basal activity in cells than PLD1^40^ and functions as both a phospholipase and as a guanine nucleotide exchange factor (GEF).^41^ The activity of PLD2 is regulated by complex phosphorylation-dephosphorylation pathways mainly on tyrosine residues^36^ through interactions with S6K, Grb2, Sos, WASp and Rac2.^42^

Surprisingly, the role of PLD1 and PLD2 in prostate cancer (PCa), the commonest cancer of men in the western world, has not been widely investigated. We have reported that PLD1 protein is preferentially expressed in basal rather than luminal prostate epithelial cell lines and in basal rather than luminal layer cells in normal prostate tissue in situ.^43^ In PCa where basal cells gradually become depleted,^44^ PLD1 protein expression is detected in the expanding population of luminal cells. PLD1 protein expression is also higher in proliferating benign prostate hyperplasia tissue compared with normal or PCa tissue.^43^ PLD activity appears not to be elevated in PCa tissue compared with normal tissue, unlike findings with other cancers (see above). PLD1 protein expression is, however, significantly higher in Gleason 7 PCa tissue compared with tissue scored Gleason 9.^43^ In this report we have investigated expression and inhibition of PLD2 in PCa using patient-derived PCa cells, prostate cell lines and tissue microarrays.

## Methods

### Prostate epithelial cell lines

The prostate epithelial cell lines used, with their growth media requirement, diagnosis and origin were as described in Noble et al.^43^ The benign hyperplasia (BPH-1) cell line^45^ was cultured in RPMI medium + 5% foetal calf serum.

### Patient-derived prostate epithelial cells

 Primary prostate epithelial cells were cultured from human prostate tissue samples, which were obtained with patient consent and full ethical approval (South Yorkshire Research Ethics Committee, Yorkshire and the Humber, REC:07/H1304/121) as previously stated.^43^ Epithelial cells were grown on collagen 1-coated 10 cm dishes in Keratinocyte serum-free medium (KSFM) with supplements of L–glutamine, bovine pituitary extract, epidermal growth factor, stem cell factor, cholera toxin, leukaemia inhibitory factor and granulocyte macrophage colony stimulating factor at 37 °C with 5% CO_2._^46,47^ Cells were initially co-cultured with irradiated (60 Gy) mouse embryonic fibroblast (STO) cells. Further subsequent passages were free of STOs and all cultures were used at the lowest practical passage number after establishment in culture (p2-p5).

### Western blotting

Epithelial cell lysates were prepared using Cytobuster Protein Extraction Reagent (71009, EMD Millipore) with protease inhibitors (cOmplete, EDTA-free Protease Inhibitor Cocktail Tablets, Roche) and PhosSTOP (Roche 04906837001). Cytoplasmic and nuclear extracts were prepared using Nucbuster (EMD Millipore 71183) following the manufacturer’s protocol. SDS-PAGE and western blotting were as described elsewhere.^48^ Primary antibodies were a rabbit anti-PLD2 antibody (PLD2-26, Denmat-Ouisse et al., 2001) used at 1:1000 and a rabbit anti-GAPDH polyclonal (Abcam ab9485) used at 1:10,000. The secondary antibody, a horse radish peroxidase (HRP)-linked anti-rabbit IgG (Cell Signalling, 7074S), was used at 1:10,000. A kaleidoscope protein ladder (Bio-Rad, 1610375) was used throughout.

### PLD inhibition and cell viability

The effects of PLD2 inhibition on the viability of prostate epithelial cell lines and patient-derived PCa cells was measured in an MTS ([3-(4,5-dimethylthiazol-2-yl)-5-(3-carboxymethoxyphenyl)-2-(4-sulfophenyl)-2H-tetrazolium) assay as described previously.^43^ The dual PLD1/PLD2 inhibitor FIPI (4-Fluoro-N-(2-(4-(5-fluoro-1H-indol-1-yl)piperidin-1-yl)ethyl)benzamide) was from Tocris. Another dual PLD1/PLD2 inhibitor 5W0 (VU0155056; N-(2-(4-(2-oxo-2,3-dihydro-1H-benzo[d]imidazol-1-yl)piperidin-1-yl)ethyl)-2-naphthamide), the PLD1 inhibitor EVJ (VU0364739; N-((S)-1-(4-(5-bromo-2-oxo-2,3-dihydro-1H-benzo[d]imidazol-1-yl)piperidin-1-yl)propan-2-yl)-2-phenylcyclopropanecarboxamide hydrochloride) and the PLD2 inhibitor JWJ (VU0364739; N-(2-(1-(3-fluorophenyl)-4-oxo-1,3,8-triazaspiro[4,5]decan-8-yl)ethyl)-2-naphtamide hydrochloride) were gifts from the late Alex Brown, Vanderbilt University, USA.^49–51^

For EVJ and JWJ in combination cells were cultured with zero, 0.25 × IC_50_, 0.5 × IC_50_, 1 × IC_50_ and 2 × IC_50_ concentrations of EVJ, JWJ and EVJ + JWJ in DMSO. See Table 1 for cell line IC_50_ values. The IC_50_ value used for patient-derived PCa cells was 6.4 μM for JWJ and 13 μM for EVJ. Cell viability was measured using an alamarBlue assay (Invitrogen Life Technologies Ltd, Paisley UK) at 24, 48 and 72 h.

**Table 1:** Inhibitor IC_50_ values for JWJ on prostate epithelial cell lines and patient-derived PCa cells compared with results for FIPI^a^, 5WO^a^ and EVJ^a^

Cell type	Inhibitor IC_50_ values (μM)			
	FIPI	5W0	EVJ	JWJ
PNT2C2	28.2	60.4	17.3	8.3
PNT1A	27.1	56.8	4.7	3.8
P4E6	13.6	10.9	9.4	4.3
LNCaP	32	24.6	14.3	12
PC3	19.3	27.3	9.8	6.3
PC3M	42.9	29.8	8	6.3
Patient 1	57.6	39.8	14.8	7.4
Patient 2	30.1	13.9	12	5.4

^a^FIPI, 5WO and EVJ data are from Noble et al.^43^

### Colony recovery assays

Patient-derived PCa cells were seeded in collagen 1-coated six-well plates at 2 × 10^5^ cells per well in complete KSFM medium. The following day cells were treated with vehicle (DMSO) or the JWJ PLD2 inhibitor (17.5 μM) for 4 h. Cultures were then rinsed, trypsinised, counted and seeded at 500 cells/well of collagen 1-coated six-well plates with STO feeder cells.^47^ The media was changed regularly and further STOs added when required. After 2–3 weeks colonies of >32 cells (at least five population doublings) were scored after being visualised by staining with 1% crystal violet in 10% ethanol in PBS.

### Immunohistochemistry

PLD2 protein expression in sections of formalin-fixed paraffin-embedded normal, BPH, PCa and CRPC (castrate-resistant prostate cancer) tissue was examined by immunohistochemistry as described previously.^43^

### Cell immunofluorescence

Cells were plated in chamber slides at 10,000 cells per well in 200 μl of media. The following day cells were fixed in 4% paraformaldehyde and rinsed with PBS. Cells were then permeabilised with 0.5% Triton X-100, rinsed, blocked (10% goat serum in PBS) and treated with primary antibody in 10% goat serum overnight at 4 °C. Next day the cells were rinsed and the appropriate Alexafluor secondary antibody added for 1 h at room temperature, followed by rinses. The chambers were removed, and the slides were mounted using Vectashield with DAPI (Vector laboratories, Peterborough, UK) and examined using a Nikon Eclipse TE300 fluorescence microscope (Nikon, Surrey, UK). The primary antibody was a rabbit polyclonal anti-PLD2 antibody (PLD2-26, Denmat-Ouisse et al., 2001) used at 1:100. The secondary antibody was a goat anti-rabbit Alexafluor 568 (A11036, Thermofisher).

### Tissue microarray (TMA) immunohistochemistry

TMAs were supplied by the Barts Cancer Institute and were immunoperoxidase-stained for PLD2 using a rabbit anti-PLD2 (PLD2-26)^52^ at 1:100 as described previously.^43^ PCa TMA1 contained 41 kidney and 168 PCa tissue sections (Gleason scores 6 (*n* = 43), 7 (*n* = 97), 8 (*n* = 13), 9 (*n* = 15). The secondary antibody was a goat anti-rabbit immunoglobulin-biotinylated (Dako E0432) used at 1:500. Tertiary antibody Streptavidin-HRP (Dako, P0397) was used at 1:100. Tissue staining was visualised using ImmPACT DAB EqV Reagent 1 and 2 from Vector Laboratories (Peterborough, UK). Stained sections on each TMA were scanned using a Zeiss AxioScan.Z1 slide scanner (ZEN 2012 software) with a Plan Apochromat ×20/0.8 objective. The composite czi files were loaded into Tissue Gnostics, GmbH, StrataQuest software (V 6.0.1.145) for analysis. The intensity of DAB staining was quantified per pixel using the workable area (mean 0.3 mm^2^) of each tissue section. Tissue was detected automatically using a combined grey image of the DAB and haematoxylin intensities; despeckle (‘Filter median’), smoothing (‘Kernel radius’) and threshold (‘Threshold CompareC’) operations were applied. Total Area Measurements of the DAB signal were extracted from the identified tissue. A scatterplot of DAB area versus DAB intensity was used to gate on intact tissue and exclude debris. Results were analysed in a GraphPad Prism statistical package (Graphpad Software, California).

### Effects of PLD inhibitors on prostate cancer cell migration

Cells were seeded on 12-well collagen-1-coated plates in complete KSFM medium and scratched with a pipette tip when 90% confluent, followed by rinsing with PBS. Treatments were then added in complete KSFM medium. Control was DMSO and PLD1 inhibitor (EVJ, VU0359595) and PLD2 inhibitor (JWJ, VU0364739) were used at 10 μM. For (A) photographs were taken at the beginning and when the Control wounds had closed. Measurements were carried out using ImageJ and are presented as % wound closure relative to control. For (B-E) Livecyte images were acquired every 15 min for 24 h. Inhibitor effects on wound closure were examined using Quantitative Phase Imaging (QPI)^53^ with the Livecyte system (Phase Focus Ltd, Sheffield, UK). This generates high contrast images and the Cell Analysis Toolbox® (CAT) software generates measurements of wound closure time, cell speed and cell direction. Analysis was carried out using the Phase Focus CAT^®^ software.

## Results

### PLD2 protein expression in PCa cells and cell lines

**Fig. 1:**
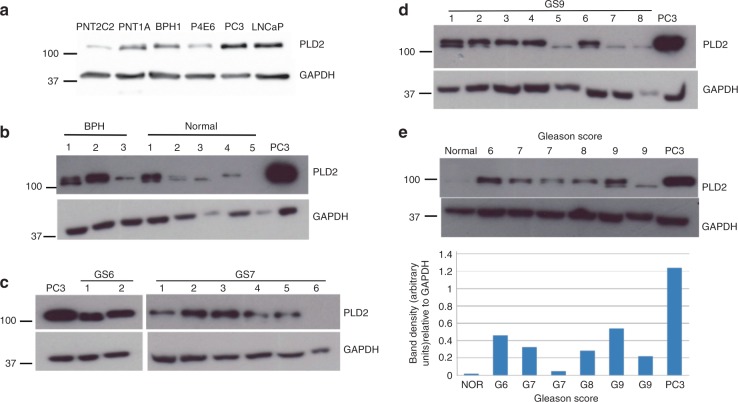
PLD2 protein expression in: **a** prostate epithelial cell lines, **b** prostate epithelial cells cultured from patient-derived benign prostate hyperplasia tissue and normal tissue, **c** cells cultured from PCa biopsies scored Gleason 6 and 7, **d** cells cultured from Gleason 9 PCa biopsy tissue compared with a PC3 positive control, and **e** prostate cells cultured from biopsies scored Gleason 6–9 and from a single biopsy scored normal with quantitation of the PLD2 band density compared with GAPDH. For all samples 20 μg protein was loaded and resolved by SDS-PAGE for western blotting with detection of GAPDH as a loading control. Markers are kDa. Blots are typical of several repeats. In **e** PLD2 band density was quantitated by Image J against GAPDH. See Materials and Methods for details

All the prostate epithelial cell lines (Fig. 1a) expressed PLD2 protein as revealed by western blotting, using a validated anti-PLD2 antibody (PLD2-26) raised by Bourgoin and colleagues.^52^ PLD2 expression was most prominent in the cancer-derived luminal LNCaP and basal PC3 cell lines. Cells cultured from five apparently normal prostate tissue biopsies showed variable PLD2 protein expression (Fig. 1b). PLD2 was also expressed in three primary prostate epithelial cell preparations (Fig. 1b) cultured from patient-derived BPH tissue. PLD2 protein was also detected in PCa cells cultured from several different Gleason-scored prostate tissue biopsies but expression varied between cell samples (Fig. 1c, d). PLD2 protein expression was generally higher in PCa cells derived from Gleason-scored cancer biopsy samples than from a tissue sample defined as non-tumorigenic (Fig.1e). In some cell samples, notably from tissue scored Gleason 9, the PLD2 band resolved into a distinct doublet (Fig. 1b, d, e).

### PLD2 protein expression in normal, BPH and PCa tissue

**Fig. 2:**
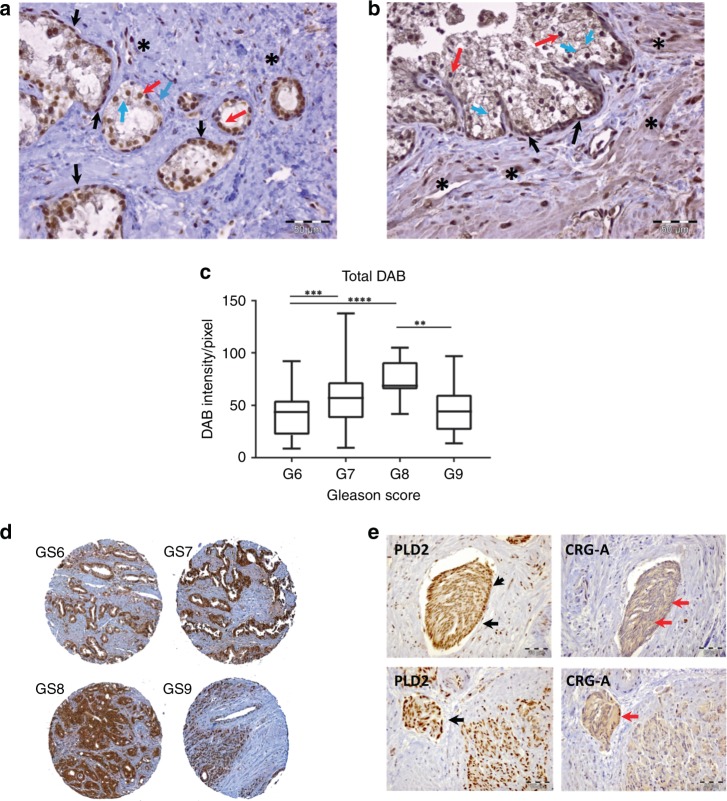
Detection of PLD2 protein in prostate tissue. **a** normal and **b** BPH. In **a**, PLD2 protein expression is detected in normal tissue in both basal (black arrows) and luminal (red arrow) prostate epithelial cells. Staining is prominent in luminal and basal cell nuclei and weaker in luminal cell cytosol and plasma membranes (blue arrows). PLD2 is also detected in occasional stromal cell nuclei (_*_). In **b**, PLD2 protein is detected in BPH tissue in basal (black arrows) and luminal (red arrows) prostate epithelial cells as well as in the stroma. Staining is prominent in luminal and basal cell nuclei, luminal cell cytosol and at luminal cell plasma membranes (blue arrows). PLD2 protein is prominent in the nuclei and cytosol of many stromal cells (_*_). Scale bar: 50 μm. **c** PLD2 protein expression was examined in a TMA of 168 PCa tissue sections, Gleason scores 6 (*n* = 43), 7 (*n* = 97), 8 (*n* = 13), 9 (*n* = 15). PLD2 expression increases significantly from Gleason score 6 tissue to Gleason 8 tissue, which is significantly higher than PLD2 protein expression in GS9 sections. **p < 0.01, ***p < 0.001, ****p < 0.0001. **d** representative Gleason 6, 7, 8 and 9 tissue sections from the PCa TMA used to give the results in **c**. Note the different tissue architecture of the Gleason 6, 7 and 8 samples compared with Gleason 9 tissue where gland structure is absent. **e** serial sections reveal that PLD2-positive cells in CRPC tissue co-express chromogranin A. PLD2 staining in sheets and foci of cells is largely nuclear with less prominent cytosolic staining (black arrows). Chromogranin A staining is largely cytosolic (red arrows). Occasional stromal cell nuclei stain strongly for PLD2. See Methods for details

In prostate tissue judged by pathology to be normal, PLD2 protein expression was detected in both basal (black arrows) and luminal (red arrows) cell layers (Fig. 2a). It was especially prominent in the nuclei of both cell layers and was weakly detected in the cytosol and at the plasma membrane (blue arrows) of luminal cells. PLD2 protein was also detected in occasional stromal cells (*). In BPH tissue (Fig. 2b) PLD2 expression levels were increased in all cell compartments compared with normal tissue (Fig. 2a). Basal cells showed prominent PLD2 staining in nuclei and cytosol as did luminal cells (black and red arrows); the plasma membrane of luminal cells was especially well defined (blue arrows). In contrast to normal tissue, PLD2 protein was also prominently expressed in the cytoplasm and nuclei of many stromal cells (Fig. 2b, *).

An increase in DAB staining indicative of PLD2 expression and measured as intensity per pixel, correlated with Gleason score up to GS8 in tissue sections on a PCa TMA (Fig. 2c). DAB intensity in the few GS9 sections analysed was significantly lower than the GS8 samples on the TMA (Fig. 2c). Typical examples of TMAs scored Gleason 6, 7, 8 and 9 are shown in Fig. 2d. PLD2 protein was detected strongly in the cytoplasm and nuclei of both basal and luminal cells in glands in Gleason 6 and 7 sections on the TMA; some PLD2 expression was also detected in occasional cells in the stroma (Fig. 2d). The poorly defined glands in sections scored Gleason 8 where luminal cells predominate also showed very prominent PLD2 protein expression. In GS9 tissue PLD2 protein was restricted to areas of small densely staining cells as shown in more detail in Supplementary Fig. 1. Stromal cells in GS9 tissue showed little PLD2 expression. Staining of near adjacent serial sections of CRPC tissue revealed that the foci of invading cells expressing PLD2 co-expressed the neuroendocrine marker chromogranin A (CRG-A) in their cytosol (Fig. 2e).

### PLD2 protein localisation in cells

**Fig. 3:**
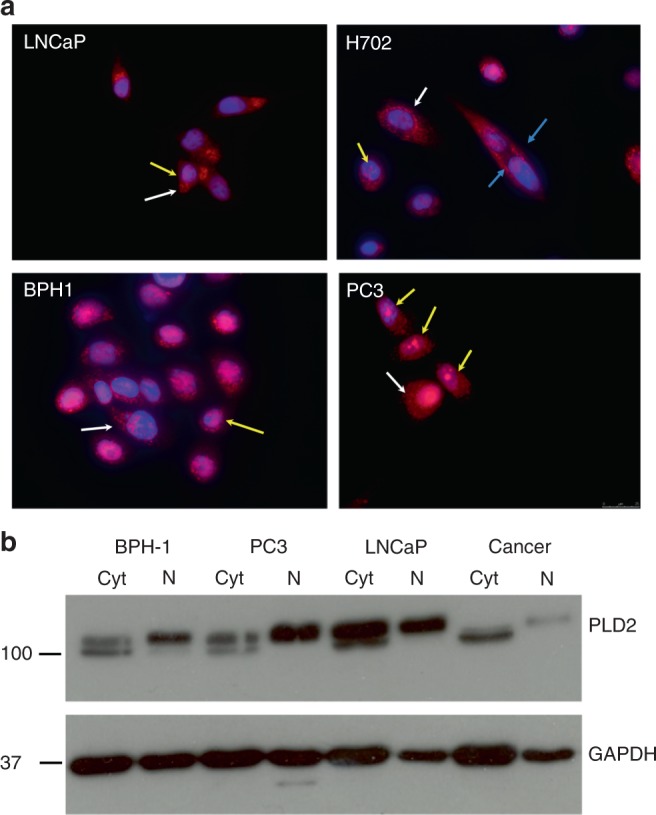
**a** Immunofluorescence detection of PLD2 in cytosol and nuclei of BPH-1, LNCaP, PC3 cell lines and PCa cells (H702). **b** Western blot of PLD2 in cytosolic and nuclear fractions purified from BPH, LNCaP and PC3 prostate cell lines and one prostate cancer tissue sample (H702). In **a**, The H702 PCa cells were purified from a prostate biopsy of Gleason score 7. In all images PLD2 has a punctate distribution in the cytoplasm (white arrows) and in nuclei as granules (yellow arrows) of varying size. In one dividing H702 cell some PLD2 may be located at the plasma membrane (blue arrows). Nuclei are defined by DAPI (mauve) and PLD2 by red. Magnification: ×60. In **b**, the cancer cells were from a biopsy scored Gleason 7. All samples, loaded as per the manufacturer’s protocol, were resolved by SDS-PAGE for western blotting with detection of GAPDH as a loading control. Markers are kDa. See Methods for details

PLD2 protein was generally detected as punctate perinuclear dots (white arrows) in the cytoplasm of BPH1, LNCaP and PC3 prostate epithelial cell lines as well as in one cancer cell preparation (H702) purified from GS7 biopsy tissue (Fig. 3a). In this one dividing PCa cell, some PLD2 appeared to be aligned to the plasma membrane (blue arrow). In all cell nuclei PLD2 protein was detected as punctate dots (speckles) or larger granules (Fig. 3a, yellow arrows); these granules were especially prominent in nuclei of PC3 cells (Fig. 3a). PLD2 remained in the nucleus when living cells were treated to remove all soluble proteins (results not shown). This cytoplasmic/nuclear distribution of PLD2 in the four cell types examined was confirmed by western blotting (Fig. 3b), which showed PLD2 protein in both the cytoplasmic and nuclear fractions. Cytoplasmic PLD2 resolved as two bands while nuclear PLD2 generally resolved as a single band corresponding to the higher molecular weight form of the cytoplasmic doublet (Fig. 3b).

### Effects of EVJ and JWJ on cell migration

**Fig. 4:**
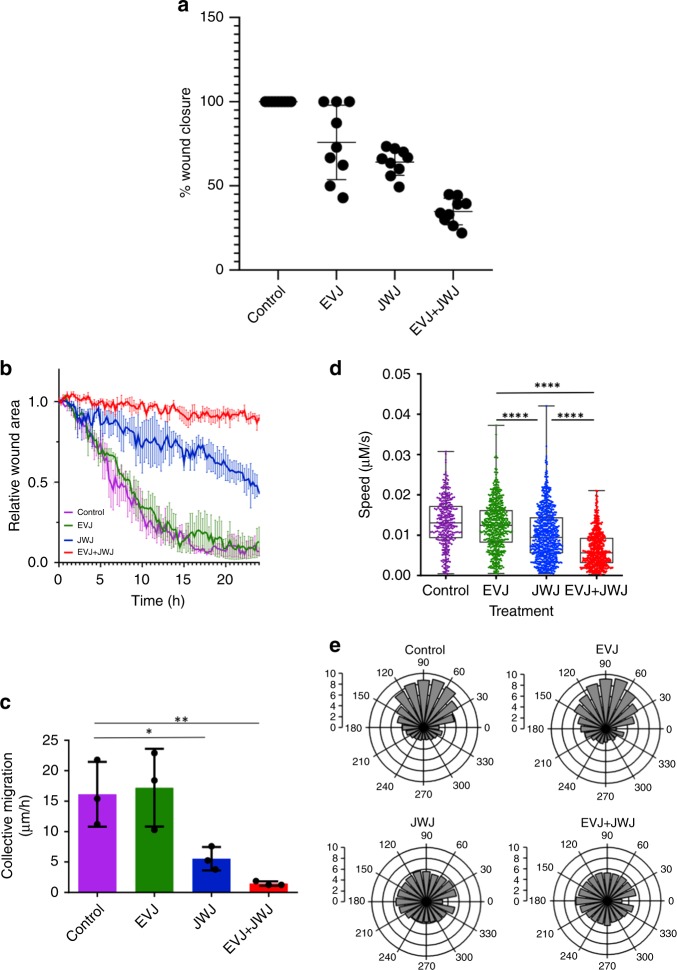
Analysis of the effects of PLD1 inhibitor EVJ and PLD2 inhibitor JWJ on prostate cancer cell wound healing. **a** wound closure time, **b** relative wound area over time, **c** collective cell migration ( μm/hr), **d** single cell speed (μm/sec) and **e** direction of cell movement. See Methodology for wound closure and QPI details. In 4 C a one-way ANOVA was carried out with Dunnett’s multiple comparisons test giving a significance of *(*p* = 0.0400) for Control vs JWJ and a significance of **(*p* = 0.0076) for Control vs EVJ + JWJ. In Fig. 4d a Kruskal-Wallis one-way ANOVA was carried out with a Dunns Multiple comparison test giving a significance of ****(*p* < 0.0001) for all comparisons apart from Control vs EVJ, which was not significant. In Fig. 4e the flat top of each segment gives the % of leading-edge cells moving in the direction shown where the centre of the rosette is the wound edge. The outer circle is 10% with inner circles reducing according to the 0–10% scales shown

Simple wound closure assays with three patient-derived cell samples in triplicate (Fig. 4a) indicated that a combination of EVJ + JWJ was more effective at inhibiting wound closure than either inhibitor alone. The JWJ inhibitor showed good grouping of results compared with EVJ where considerable patient variability was observed. Further insight into the effect of these inhibitors on wound closure was gained by QPI analysis in triplicate with cells from one biopsy sample. This revealed that the PLD2 inhibitor JWJ delayed wound closure more effectively than the PLD1 inhibitor EVJ (Fig. 4b), which hardly differed from the DMSO control. A combination of EVJ + JWJ (10 μM each) slowed wound closure even more as also revealed by analysis of the collective migration output (Fig. 4c). QPI also showed that JWJ was more effective than EVJ at reducing the rate at which single PCa cells at the leading edge moved after wounding; again, even more effective inhibition of movement occurred when EVJ and JWJ were applied together (Fig. 4d). QPI analysis allowed a determination of the direction in which cells at the leading edge of the wound were moving. In Fig. 4e each segment represents the percentage of leading-edge cells moving in the direction shown. DMSO-control and EVJ-treated cells were mostly all moving away from the wound edge (0–180^o^ axis) into the gap to close the wound while cells treated with JWJ or JWJ + EVJ migrated more randomly at the leading edge. Many JWJ- or JWJ/EVJ-treated cells moved back into the cell layer (Supplementary Video 1).

### PLD inhibitor effects on cell viability and colony formation

**Fig. 5:**
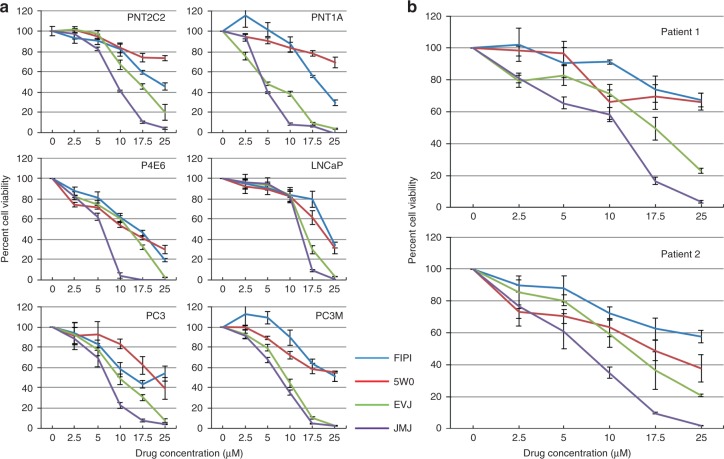
Effect of the dual PLD1/PLD2 inhibitors FIPI, 5WO, the specific PLD1 inhibitor EVJ and the specific PLD2 inhibitor JWJ on prostate epithelial cell viability. **a** prostate cell lines, **b** patient-derived PCa cells. Cells were cultured with 0–25 μM concentrations of FIPI, 5W0, EVJ and JWJ dissolved in DMSO. Cell viability was measured by MTS assay after 48 h. Results are expressed as percentage cell viability relative to DMSO controls. See Methods for details

**Fig. 6:**
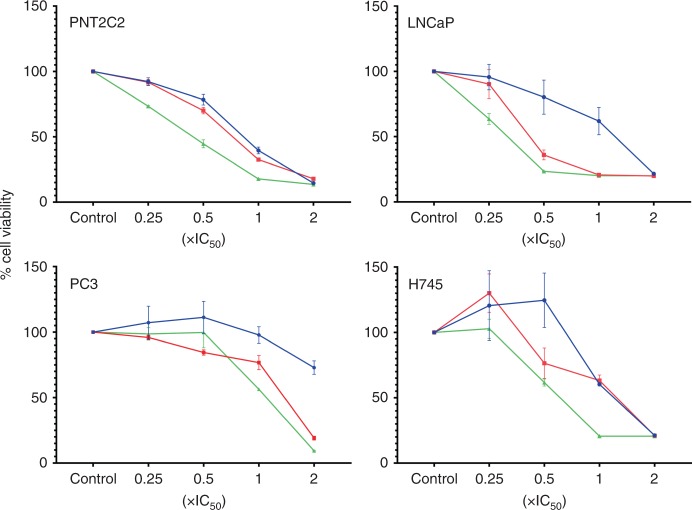
Effect of inhibiting PLD1 and PLD2 together on the viability of prostate cell lines and patient-derived PCa cells. Cells were cultured with zero, 0.25 × IC_50_, 0.5 × IC_50_, 1 × IC_50_ and 2 × IC_50_ concentrations of EVJ + JWJ in DMSO (green lines). The IC_50_ value used for patient-derived PCa cells was 6.4 μM for JWJ (red lines) and 13 μM for EVJ (blue lines). See Table 1 for cell line IC_50_ values. Results shown are for 72 h and are expressed as percentage cell viability relative to DMSO controls. Error bars at each concentration point are +/− the standard deviation, *n* = 3. Two other PCa cell preparations (H741 and H742) gave similar results to that shown for H745. See Methods for details

When cell viability was assessed, the specific PLD2 inhibitor JWJ was much more effective (Fig. 5a) than the dual PLD1/PLD2 inhibitors FIPI or 5WO and even the PLD1 inhibitor EVJ as reported previously.^43^ At a concentration of 17.5 μM the viability of all PCa cell lines was reduced to almost zero after 48 h treatment. The cell lines PNT1A and P4E6 seemed especially sensitive to the effects of JWJ where maximal effect occurred at a concentration of 10 μM. JWJ also reduced the viability of patient-derived epithelial cells (Fig. 5b) more effectively than FIPI, 5WO or EVJ. This is confirmed by the cellular IC_50_ values, which were calculated using GraphPad prism (Table 1). Data for FIPI, 5W0 and EVJ are from Noble et al.^43^ and are included in Table 1 and Fig. 5 for comparison. Application of JWJ and EVJ in combination (Fig. 6) inhibited the viability of prostate epithelial cell lines PNT2C2, LNCaP and PC3 and patient-derived PCa cells more effectively than when used alone. Two other PCa cell preparations gave similar results to that shown for H745.

Exposure of patient-derived prostate cancer cells to 17.5 μM JWJ for 4 h significantly reduced subsequent cell colony formation (>32 cells) relative to DMSO vehicle controls (Supplementary Fig. 2). JWJ had a significantly greater inhibitory effect on colony formation than EVJ as the median reduction, relative to controls by EVJ, was about 30% compared with 50% for JWJ. Data for EVJ are from Noble et al.^43^ and are included in Supplementary Fig. 2 for comparison.

## Discussion

A unique feature of this initial study on the role of PLD2 in PCa is the use of patient-derived cells cultured from Gleason-scored biopsies. These have a basal phenotype^46^ and allow us to approximate the *in vivo* state as closely as possible, compared with using immortalised prostate cell lines. The results indicate that (1), unlike PLD1 expression luminal and basal PCa cells express PLD2 protein about equally, (2), PLD2 regulates PCa cell proliferation and colony formation, (3), PLD2 is involved in directed cell migration in PCa cells, (4), PLD2 protein expression increases with PCa Gleason scores from 6 to 8, (5), in BPH tissue stromal cells as well as basal and luminal cells show upregulated PLD2 expression and (6) intriguingly, PLD2 protein is co-expressed with chromogranin A (a neuroendocrine marker) in CRPC tissue. Our biopsy samples come with Gleason scores using the 2005 ISUP grading system.^54^ These can be converted to the newer 2014 five grade grouping as detailed in Berney et al.^55^

### PLD2 protein expression in prostate cells, tissue and PCa TMAs

We tested several commercial anti-PLD2 antibodies but reproducible western blot results giving a single band (and occasionally a doublet) of the correct molecular size were only obtained with a validated anti-PLD2 antibody, PLD2-26 of Denmat-Ouisse et al.^52^ Our western blot findings that both cancer-derived basal PC3 and luminal LNCaP cell lines show similar levels of PLD2 protein expression agree with recent findings of Utter et al.^10^ This expression pattern is, however, quite distinct from that of PLD1, which we found to be expressed predominantly in basal prostate epithelial cell lines and in basal layer cells in situ.^43^ This difference is confirmed by our IHC results, where PLD2 protein is detected in both basal and luminal layer cells in glands of tissue identified as normal (Fig. 2a) or BPH (Fig. 2b). Compared with PLD2, PLD1 has low intrinsic activity in cells and requires activation.^40,56^ Therefore, in basal layer cells its activity will be regulated by stromal factors such as FGF and TGFβ diffusing through the basal lamina.^57,58^ The upregulated PLD2 expression detected in stromal cells in BPH tissue compared with normal stroma is probably due to invading immune cells and/or activation of PLD2 expression in smooth muscle cells and fibroblasts resulting from the inflammatory processes characterising this condition^59^ (often termed cancer-reactive stroma or cancer-associated fibroblasts, CAF). Basal PCa epithelial cells purified from BPH and PCa biopsies express PLD2 protein (Fig. 1b, c, d); the observed variation in expression most probably arises from inter-patient variability. The western blot finding that PLD2 protein expression is greater in PCa cells purified from biopsies scored Gleason 6–9 compared with cells from normal biopsy tissue (Fig. 1e) implies that PLD2 expression is increased in PCa, as has been reported for renal, colon, colorectal and other human cancers.^4,5,60,61^ This was not observed for PLD1^43^ but the result for PLD2 is supported by our IHC analysis of a PCa TMA (Fig. 2c, d) where the intensity of DAB reaction product/pixel increases significantly in tissue sections scored Gleason 6–8 but is lower in Gleason 9 sections. This finding suggests that PLD2 is more actively involved in the early development of PCa when luminal cells are proliferating in glands rather than when gland structure has disappeared and tumour cells are present in nests and sheets infiltrating the stroma (Fig. 2d, Supplementary Fig. 1). In CRPC tissue invading PLD2-positive PCa cells co-stain for chromogranin A (Fig. 2e), an indicator of the development of aggressive androgen-independent neuroendocrine PCa^62–64^ through Akt/hnRNPK/AR/β-catenin^65^ and/or N-Myc-driven^66^ pathways. These PLD2-positive PCa cells in CRPC also express PLD1.^43^

### PLD2 localisation

Our IF results (Fig. 3) indicating that PLD2 protein is located in both the cytosol and nuclei of prostate epithelial cell lines are confirmed by the IHC results on sections of normal prostate tissue (Fig. 2a) where nuclei and cytosol in both luminal and basal layer cells are positive for peroxidase reaction product. PLD2 in the cytosol has a punctate perinuclear distribution like that of PLD1 in prostate basal cells,^43^ and similar to that reported for PLD2 in other cells.^30,52,67^ These results support the prevailing view that PLD2 (like PLD1) has a role in membrane vesicle transport to and from the Golgi complex.^68^ Supporting this conclusion are findings that PtdOH generated by PLD2 is involved with BARS protein in COP1 vesicle fission,^69^ with Golgi tubule formation and Arf GAP1 recruitment^70^ and in continuous vesicle movement from the cell membrane to the nucleus.^71^ PLD2 localisation on endosomal/exosomal structures is well documented.^72^ Our IF results indicate that PLD2 is not, however, usually located at the plasma membrane in prostate epithelial cell lines and PCa cells under the experimental conditions used. One rare exception is a dividing PCa cell, in which some PLD2 may be aligned at the plasma membrane (Fig. 3, H702, blue arrow). Generally therefore, our IF results on PLD2 localisation agree with Freyberg and Iyer^30,67^ but contrast with other reports.^24,73–76^ We concur with the views of Frohman and colleagues^76^ that PLD2 localisation in cells most likely varies according to cell type, activation state and perhaps also to stage of cell division. In support, PLD2-immunoreactive staining at the plasma membranes of luminal cells in BPH tissue sections (Fig. 2b, blue arrows) is much more pronounced than in luminal cells in normal tissue stained under identical conditions (Fig. 2a, blue arrows). This suggests that in BPH some PLD2 locates to the plasma membrane perhaps through interaction with the EGF receptor,^26^ which shows increased expression in BPH.^77^ A similar effect might be expected to occur in PCa tissue where the EGF receptor is overexpressed and correlates with disease progression.^78,79^ Such a translocation is observed in renal cancer cells, which show increased PLD2 staining at the plasma membrane compared with normal cells.^4^ Interestingly, the EGF receptor is transported from the nucleus to the plasma membrane in PtdOH-recycling vesicles.^71^

### PLD2 in the nucleus

Our IF, IHC and western blot results all indicate that some PLD2 protein is present in the nucleus of the various prostate cells and tissue sections studied. Cytosolic PLD2 resolves as a doublet in some western blots (Fig. 3b). The lower band in this doublet is unlikely to be a non-specific band of similar molecular weight to PLD2 as found by Bruntz et al.^13^ using a commercial anti-PLD2 antibody since, if non-specific, it would be detected in every lane and this is not observed. Thus, this doublet in the cytosol may reflect differences in PLD2 phosphorylation on tyrosine or serine/threonine residues.^34,36,80,81^ In support, others have reported that tyrosine phosphorylation of PLD2 induces a band shift.^82^ Interestingly, nuclear PLD2 generally resolved as a single band corresponding to the upper band of the cytosolic doublet. Whether this higher molecular size form of PLD2 shuttles into the nucleus from the cytosol is not clear. PLD1 has a nuclear localisation signal in its loop region, which interacts with β-importin^18^ to promote nuclear translocation. However, this loop region, which accounts for the low basal activity of PLD1,^56^ is missing in PLD2. Yet PLD2 can move into the nucleus as occurs after brefeldin-A treatment of cells,^30^ for example. It is interesting that PtdOH, the product of PLD2 activation, can assist the nuclear import of proteins that lack a classical nuclear localisation signal.^83,84^

Some of the PtdIns(4,5)P2 needed for PLD2 activity exists in the nucleus^85^ where it is mostly associated with proteins as detergent-insoluble proteolipid complexes.^85,86^ These complexes appear as speckles and granules very similar in appearance to the spots of nucleoplasmic PLD2 we observe in PCa cells (Fig. 3a). PtdOH formed by nuclear PLD2 activates phosphatidylinositol-4 P5-kinase^87^ a component of the nuclear speckles together with pre-mRNA splicing factors. This points to a role for PtdOH (and therefore PLD) in pre-mRNA splicing or mRNA metabolism and export. PtdOH generated in the nucleus by PLD2 may also regulate cell proliferation by activating nuclear mTOR^88–91^ and/or nuclear ERK.^92^ Such results explain why we find that inhibiting PLD2 has a marked effect on PCa cell proliferation and colony forming ability, as discussed below. PtdOH also regulates nuclear/cytoplasmic shuttling^83^ and is an intermediate in phospholipid biosynthesis.^17^ Interestingly, nuclear PLD1 in vascular smooth muscle cells is activated by cell surface G-protein-coupled receptors via PI3K, PKCζ and/or RhoA pathways, but not by activation of receptor tyrosine kinases.^93^ Whether nuclear PLD2 and PLD1^43^ in PCa cells is regulated similarly is as yet unknown.

### Prostate cell migration

Both methods of analysis used indicate that PLD2 has a more significant role in controlling PCa cell migration than PLD1 (Fig. 4a–d). This agrees with findings for the PC3 prostate epithelial cell line^10^ and is probably due to the fact that PLD2 is intrinsically active in cells, while PLD1 requires activation.^40^ However, PLD1 apparently plays some role in PCa cell migration since wound closure time lengthens when PLD1 and PLD2 are inhibited together. This could be because PLD1 can control cell-substratum interactions through a lipase-independent Src/Pyk2 pathway.^94^ More detailed analysis by QPI reveals that inhibiting PLD1 with EVJ or PLD2 with JWJ significantly reduces the rate of PCa cell movement (Fig. 4d). However, this is not the main reason why wound closure by JWJ-treated PCa cells is delayed compared with control and PLD1-inhibited cells. Inhibiting PLD2 appears to cause leading-edge cells to move in a random chemokinetic manner rather than in the directed chemotactic migration of control and PLD1-inhibited cells across the wound (Fig. 4e). PLD2-inhibited cells still migrate, albeit slower and randomly, so any PtdOH required to stabilise mTOR for cell motility^95–97^ is probably being generated by lysophosphatidic acid acyltransferase (LPAAT) and/or diacylglycerolkinase (DAGK) pathways^19^ and/or by a Grb2, Rac2 and WASp pathway involving the PX and PH domains of PLD2.^42,98^ Control and PLD1-inhibited cells migrate in a directed manner because they respond normally via cell surface receptors to chemoattractant signals such as Ca^2+^ and nucleotides generated during wounding.^99–102^ Currently we do not know why inhibiting PLD2 causes PCa cells to lose their sense of direction. PLD2 is connected with the production of PtdIns 3,4,5-P3 (PIP3), the level of which is regulated by phosphoinositide-3-kinase (PI3K) and the phosphatase and tensin homologue (PTEN)^103^ both of which are key players of directional sensing in eukaryotic cells;^104^ PIP3 is enriched on the potential anterior side of migrating cells. Inhibiting intrinsically active PLD2 may reduce PtdOH levels in the plasma membrane needed with PtdIns 3,4,5-P3 to stabilise atypical GEFs such as dedicator of cytokinesis DOCK2 or DOCK180 as occurs in neutrophils or epithelial cells, respectively.^105,106^ DOCK2 and DOCK180 activate Rac involved in organising membrane extensions in the direction of migration and absence of PtdOH results in abnormal leading edges and defective chemotaxis.^105^ In neutrophils Rac1 is critical for gradient detection and orientation toward a chemoattractant source while Rac2 is the main regulator of actin assembly and migration.^107^ It remains to be discovered whether DOCK proteins and Rac1 are similarly involved in the failure of PLD2-inhibited PCa cells to show directed migration. Interestingly, inhibiting PLD2 in breast cancer cells can block metastasis because PtdOH is unavailable to bind the motor protein KIF5B, which controls membrane trafficking of the MT1-matrix metalloproteinase needed for invadopodia formation and invasion.^108^ Reduced levels of PtdOH in PCa cells by inhibition of PLD2 could also interfere with migration through integrin activation and the formation of stable adhesions.^109^

### Inhibitor effects on cell proliferation

Since PLD2 regulates nuclear ERK activity in several cancer cell lines^92^ it is not surprising that proliferation of prostate cell lines and patient-derived PCa cells is effectively inhibited by JWJ (Fig. 5a, b). However, it is somewhat surprising that inhibiting PLD2 with JWJ reduces cell viability to zero in these cells when compensatory pathways for the formation of PtdOH including LPAAT and DAGK exist^19^ and when knocking out PLD2 in mice is not lethal.^110,111^ As with PLD1^31^ the effect of PLD2 on nuclear ERK might involve activation of PKCα.^33^ Our IC_50_ results (Table 1) suggest that prostate cell proliferation is more sensitive to PLD2 inhibition than to PLD1, which may be attributed to its dual role as a GEF as well as a phospholipase.^36,42^ Though we used different concentrations of inhibitors our EVJ + JWJ combination results agree with Utter et al.^10^ that the viability of luminal LNCaP cells is more sensitive to inhibition of PLD1 and PLD2 together than the basal PC3 cell line. The fact that a combination of EVJ + JWJ is more effective at inhibiting the viability of basal patient-derived PCa cells than when inhibitors are used singly, emphasises that PLD1 and PLD2 must work together in the maintenance of PCa cell viability as is also observed for receptor-mediated endocytosis^112^ and mTOR activation.^113^ The effectiveness of these inhibitors is emphasised by our colony formation finding (Supplementary Fig. 2) that even short-term inhibition of PLD2 (and PLD1^43^) has long term effects on PCa cell viability. We have explained previously^43^ why our IC_50_ values for PLD1/2 inhibition of prostate cell proliferation are higher than reported by others.^12,50,114^ These specific PLD1 and PLD2 inhibitors are based on the antipsychotic agent halopemide, which has been tested in clinical trials and is well tolerated.^115,116^ Our results in this report show that these PLD1 and PLD2 inhibitors have considerable potential for treating PCa especially if used in combination.

## Supplementary information

Supplementary Figures and legends

Wound Healing

## Data Availability

The data used in this article are stored securely at the University of York and are available on request.
