# Supplementary material for: Phospholipase D2 in prostate cancer: protein expression changes with Gleason score
Source: Br J Cancer. 2019 Nov 1;121(12):1016–26. doi: 10.1038/s41416-019-0610-7 (PMC6964697; doi:10.1038/s41416-019-0610-7)
Supplement: Supplementary file 1 — Supplementary Figures and legends [file 41416_2019_610_MOESM1_ESM.pptx]

## Slide 1
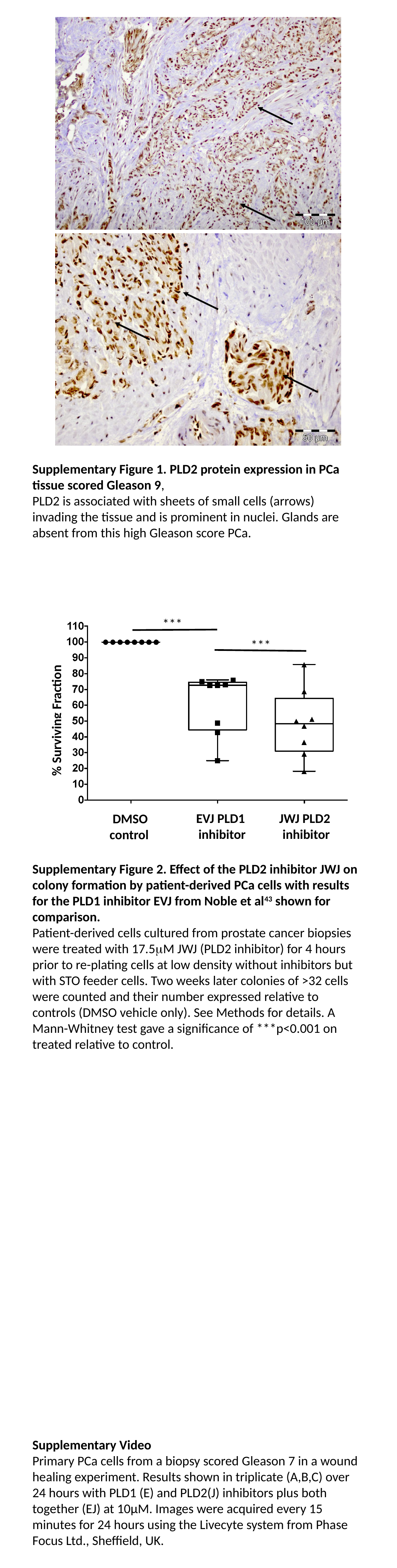

B
Supplementary Figure 1. PLD2 protein expression in PCa tissue scored Gleason 9,
PLD2 is associated with sheets of small cells (arrows) invading the tissue and is prominent in nuclei. Glands are absent from this high Gleason score PCa.
***
***
% Surviving Fraction
Control
JWJ PLD2
inhibitor
EVJ PLD1
inhibitor
 DMSO
control
Supplementary Figure 2. Effect of the PLD2 inhibitor JWJ on colony formation by patient-derived PCa cells with results for the PLD1 inhibitor EVJ from Noble et al43 shown for comparison.
Patient-derived cells cultured from prostate cancer biopsies were treated with 17.5mM JWJ (PLD2 inhibitor) for 4 hours prior to re-plating cells at low density without inhibitors but with STO feeder cells. Two weeks later colonies of >32 cells were counted and their number expressed relative to controls (DMSO vehicle only). See Methods for details. A Mann-Whitney test gave a significance of ***p<0.001 on treated relative to control.
Supplementary Video
Primary PCa cells from a biopsy scored Gleason 7 in a wound healing experiment. Results shown in triplicate (A,B,C) over 24 hours with PLD1 (E) and PLD2(J) inhibitors plus both together (EJ) at 10μM. Images were acquired every 15 minutes for 24 hours using the Livecyte system from Phase Focus Ltd., Sheffield, UK.
